# A meta-analysis of cohort studies: Traumatic brain injury and risk of Alzheimer’s Disease

**DOI:** 10.1371/journal.pone.0253206

**Published:** 2021-06-22

**Authors:** Jieyu Zhang, Yongkang Zhang, Juntao Zou, Fei Cao

**Affiliations:** 1 Fuzhou Medical College of Nanchang University, Fuzhou, China; 2 Jiangxi Cancer Hospital, Nanchang, China; Istituto Di Ricerche Farmacologiche Mario Negri, ITALY

## Abstract

**Introduction:**

Recently, some epidemiological studies have reported that cognitive disorders in elderly people is accelerated with traumatic brain injury. But the causal relationship between traumatic brain injury and AD is still an area of controversy.

**Aims:**

Our review was conducted to estimate the relation between traumatic brain injury and risk of AD.

**Methods:**

All longitudinal population-based studies comparing incidence of AD between subjects with and without traumatic brain injury from their inception to September 2020 were searched in The Cochrane Library, PubMed, Medline, Embase, Web of Science without restriction of language. The meta-analysis was conducted using Stata software.

**Results:**

A total of 17 studies involving 4289,548 individuals were included. After pooling these 17 studies, subjects with traumatic brain injury had significant higher incidence of AD than those without traumatic brain injury (RR: 1.17, 95% CI: 1.05–1.29). When considering the severity of traumatic brain injury, this elevated risk of AD was still significant comparing subjects with moderate and severe traumatic brain injury and those with no traumatic brain injury (RR: 1.30, 95% CI: 1.01–1.59).

**Conclusion:**

Traumatic brain injury, especially moderate and severe traumatic brain injury may be associated with increased risk of AD.

## Introduction

Traumatic brain injury is a common injury sustained through military service, sports involvement, falls, or other accidents. As a leading cause of death and disability in individuals aged <45 years, it is a significant public health problem associated with both acute and long-term disabilities [1]. Sixty-nine million individuals are estimated to suffer TBI from all causes each year [2].

As a progressive, neurodegenerative disease, AD is characterized by cognitive disorder, dementia, and memory loss. It is pathologically defined by the accumulation of intracellular neurofibrillary tangles and extracellular amyloid plaques in the brain [3–6]. Autopsies of relatively young TBI patients who died during the acute phase after injury show diffuse amyloid plaques and neurofibrillary tangles similar to those found in AD patients located in the areas surrounding the lesion sites in both gray and white matter regions [7]. So the connection between traumatic brain injury and AD has attracted a great deal of research interest.

Recently many observational studies [8–10] have attempted to explore the relationship between traumatic brain injury and AD, but the results of different studies are controversial.

In this review, we gathered data from all cohort studies of traumatic brain injury and AD, and a meta-analysis containing 4289,548 individuals was conducted to investigate the evidence for or against traumatic brain injury as a risk of AD.

## Materials and methods

### Data sources

The following electronic databases were searched from their inception to September 2020: The Cochrane Library, PubMed, Medline, Embase, Web Science. We included all longitudinal population-based studies of traumatic brain injury and AD without any restriction of language, and the following keywords were included: Alzheimer’s disease, dementia, AD, cognitive decline, head injury, traumatic brain injury, brain injury.

### Inclusion and exclusion criteria

Included studies met the following criteria: (i) cohort studies, (ii) head injury or traumatic brain injury as the exposure factor, (iii) the incidence of AD could be compared between subjects with and without traumatic brain injury, (iv) at baseline, subjects with any dementia were excluded. We excluded studies met the following criteria: (i) review and meta analyses, (ii)other types of dementia, (iii) there were insufficient data to estimate a relative risk (odds ratio, risk RR or hazard ratio), (iv) overlapping with other studies or overlapping with data from the same authors.

### Quality assessment

Two authors independently assessed risk of bias of the potentially included studies according to the Newcastle-Ottawa Scale [11], and discrepancies were resolved by discussion. Risk of bias of every study included the following domains: representativeness of the exposed cohort, selection of the non exposed cohort, ascertainment of exposure, demonstration that outcome of interest was not present at start of study, comparability of cohorts on the basis of the design or analysis, assessment of outcome, was follow-up long enough for outcomes to occur and adequacy of follow up of cohorts. We identified ‘high’ quality choices with a ‘star’. A maximum of one ‘star’ for each item within the ‘Selection’ and ‘Outcome’ categories, but maximum of two ‘stars’ for ‘Comparability’ were prescribed. We categorized star-earning responses in the Newcastle-Ottawa Scale as “low risk of bias” and all other responses as “high risk of bias.”

### Data extraction

Two researchers reviewed and screened independently the titles and abstracts to identify eligible trials according to the inclusion criteria, the full text was read if necessary. Disagreements were resolved by a consensus process. Included studies were extracted the data on name of the first author, publication year, study design, follow-up time in years, number of patients in the analysis, sex of patients, age of patients, method of AD assessment, overall incidence of AD, RR of AD in participants with traumatic brain injury.

### Traumatic brain injury severity

A variety of criteria exist to define TBI severity. In our study TBI was coded as mild (ICD-10 code S060, ICD-8 and ICD-9 code 850) or moderate and severe (ICD-10 code S06x, excluding S060, ICD-8 and ICD-9 code 851).

### Statistical analysis

The risk ratio (RR) was calculated by dividing the “risk of AD in exposed group” by the “risk of AD in nonexposed group”. Data were entered into Stata meta-analysis program (Stata 12.0, StataCorp, College Station, Texas). We calculated the incidence of AD before pooling. We estimated the percentage of variability contribution to heterogeneity with the *I*^2^statistic. All p values were calculated from two-tailed tests of statistical significance with a type I error rate of 5%. Egger’s test was used to assess the publication bias.

## Results

### Characteristics of studies

**Fig 1:**
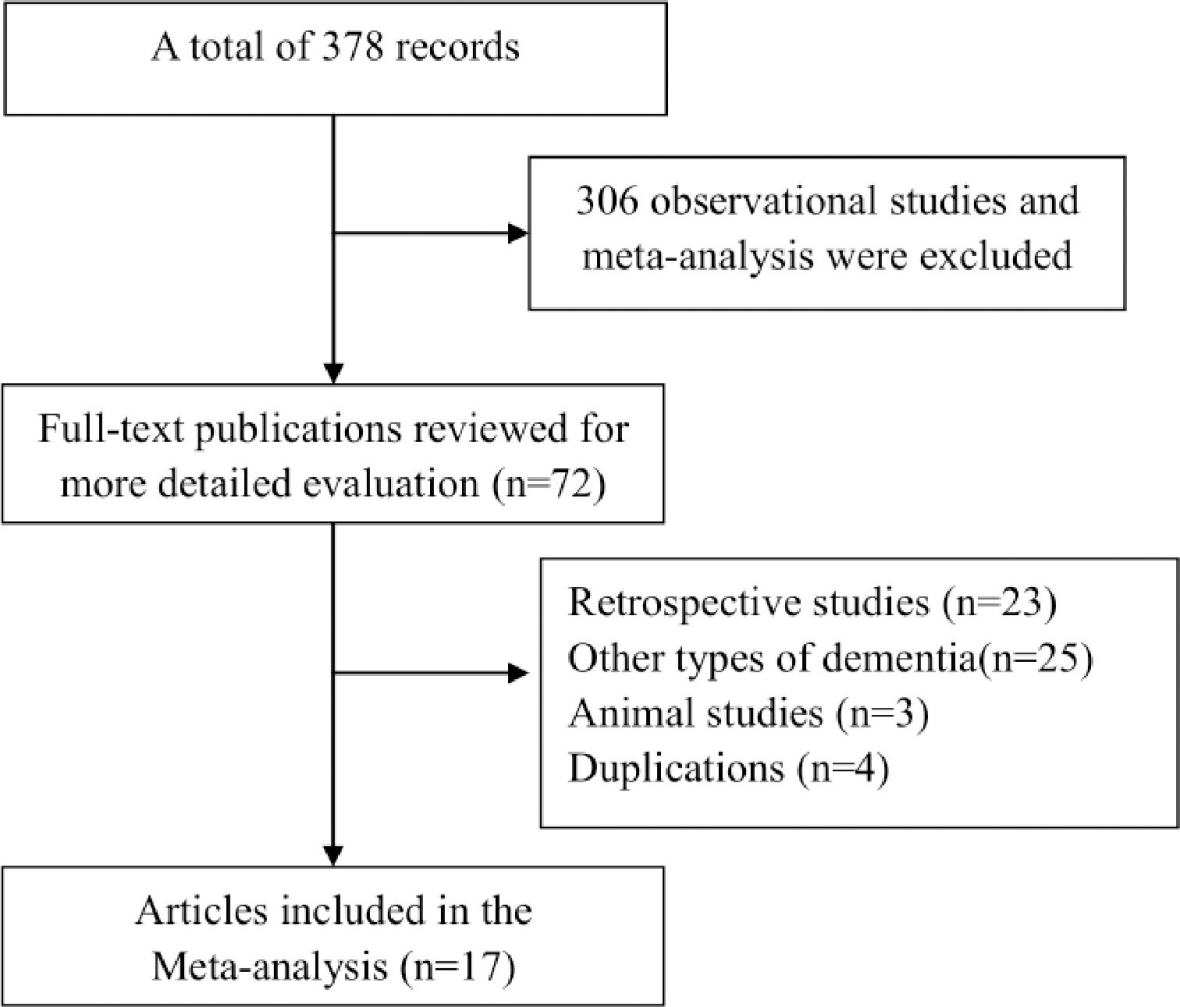
Flow chart of study selection process.

A total of 378 potentially individual titles and abstracts were found from The Cochrane Library, PubMed, Medline, Embase, Web Science. But only 72 studies were preliminarily identified and went forward to the data extraction stage. Then reviewing the full-texts of those studies, 17 studies were eligible for inclusion and 56 studies were excluded. Among these excluded studies, 4 were duplications, 3 were animal studies, 25 were excluded for studies assessing other types of dementia, 23 were retrospective studies. Seventeen cohort studies with a total of 4289,548 individuals were finally included into the meta-analysis (see Fig 1). The main characteristics of those 17 studies were listed in S1 Table.

### Quantitative data synthesis

**Fig 2:**
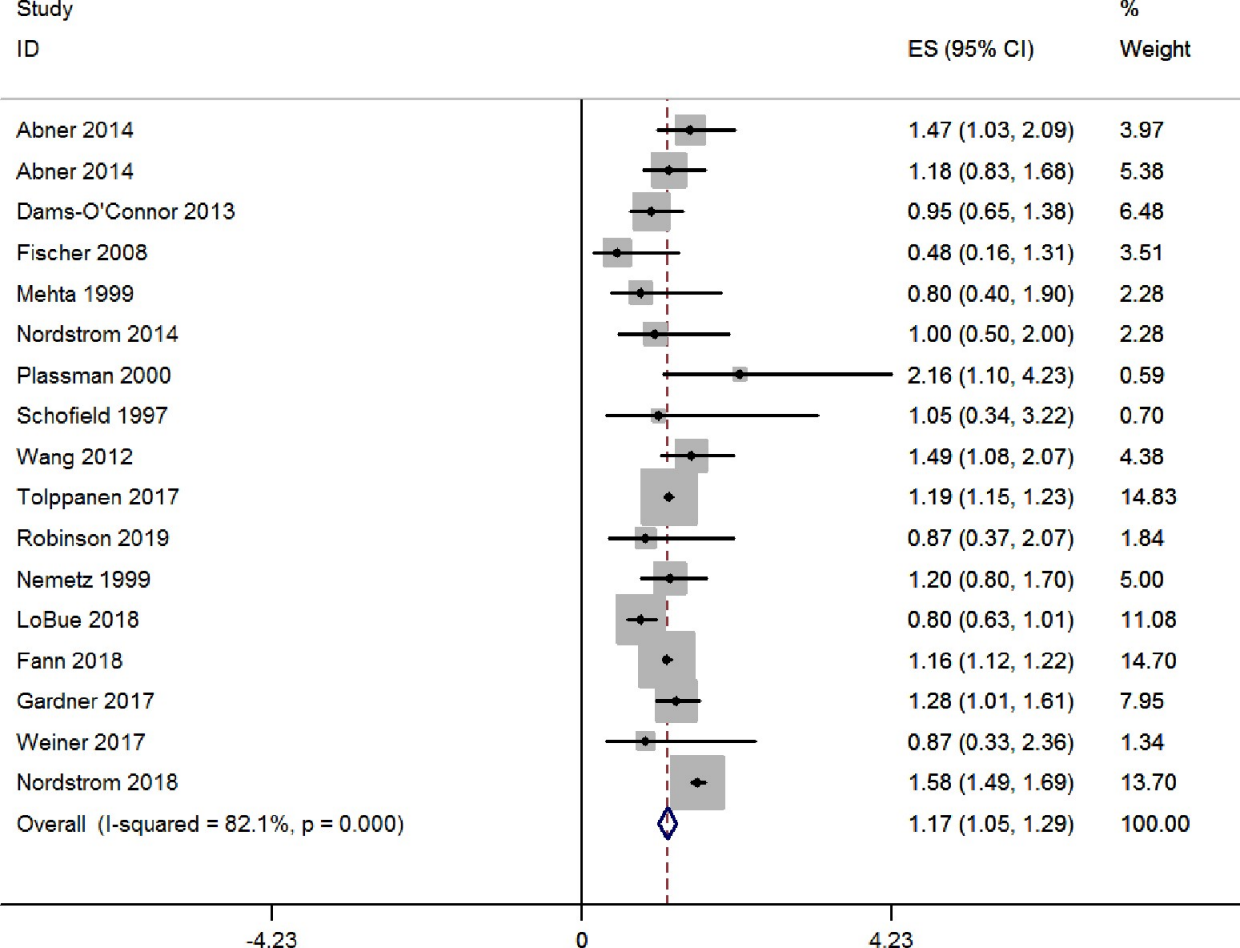
Forest plot of traumatic brain injury and the risk for AD.

In the sixteen studies comparing incidence of AD between subjects with and without traumatic brain injury, ten studies showed that traumatic brain injury could not increase the risk for AD, but six studies showed traumatic brain injury as a significant risk for AD. There were 710,858 and 1,035,919 subjects with and without traumatic brain injury respectively. After pooling these 17 studies, subjects with traumatic brain injury had significant higher incidence of AD than those without traumatic brain injury (RR: 1.17, 95% CI: 1.05–1.29) (see Fig **2**).

**Fig 3:**
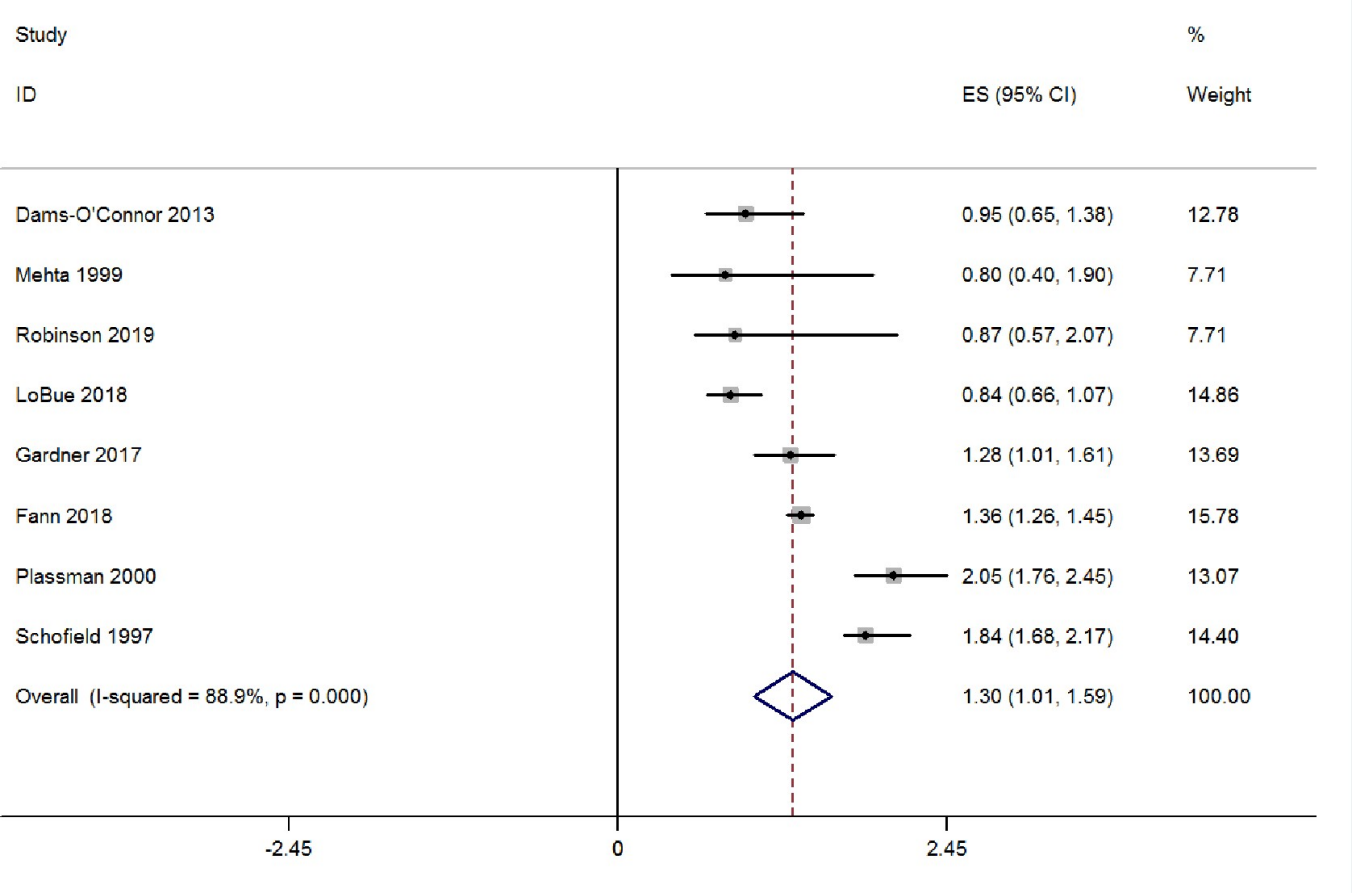
Forest plot of moderate/severe traumatic brain injury and the risk for AD.

When considering the severity of traumatic brain injury, eight studies defined the severity of traumatic brain injury. The relative risk of AD in moderate and severe traumatic brain injury populations was 1.30 (1.01–1.59) (see Fig **3**).

### Test of heterogeneity and publication bias

**Fig 4:**
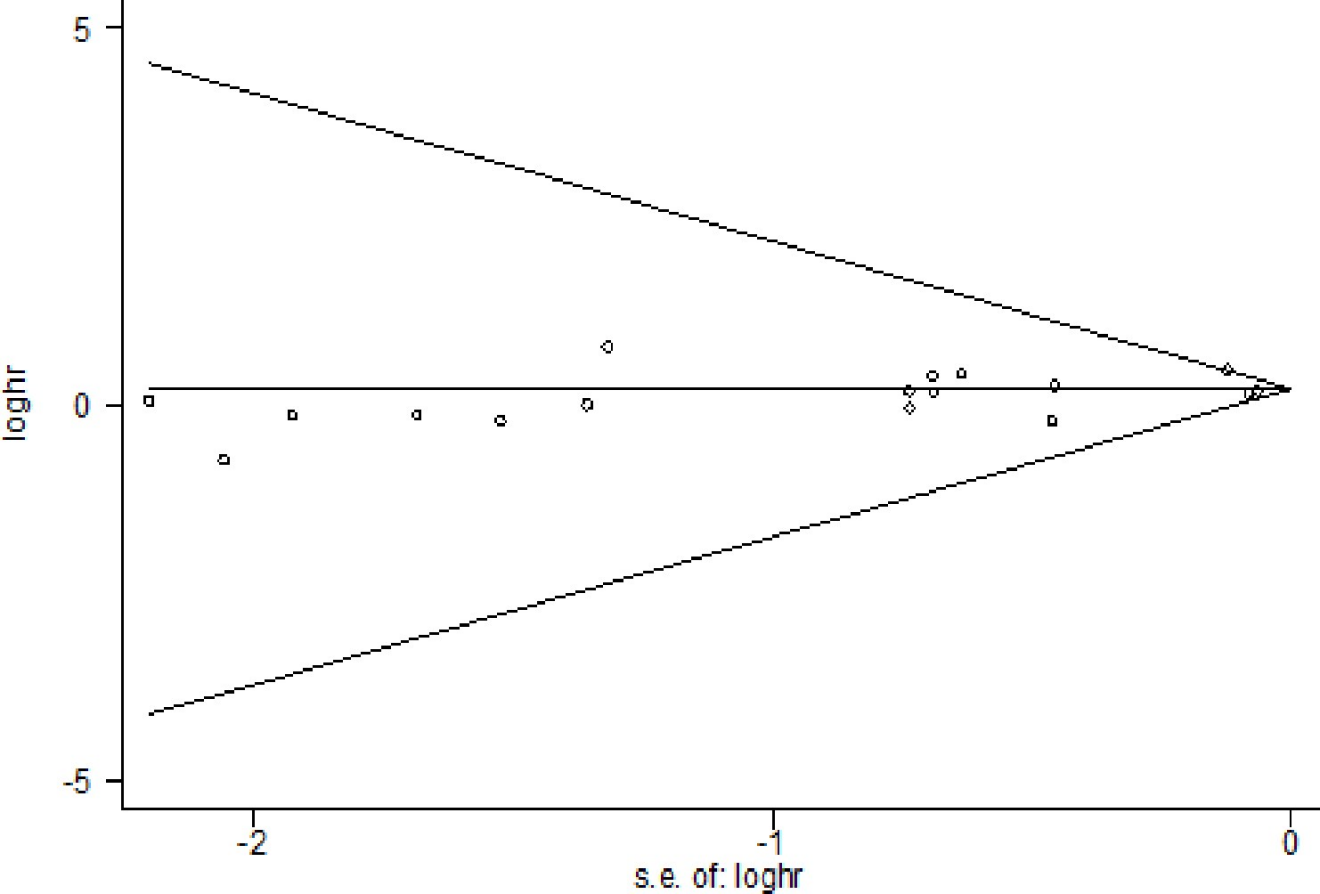
Funnel plot of traumatic brain injury and the risk for AD.

For the overall meta-analysis, the *I*^*2*^ statistic for heterogeneity among seventeen studies was 82.1%, suggesting potential between-study heterogeneity. However, the *I*^*2*^ statistic among studies reporting moderate and severe head injuries was 88.9%. A funnel plot was used to assessed publication bias (see Fig **4**). The reasonably symmetrical shape did suggest the absence of publication bias (Begg’s test *P* = 0.77).

## Discussion

In our study, pooling 17 cohort studies, subjects with traumatic brain injury had significant higher incidence of AD than those without traumatic brain injury. The finding reinforces the evidence that traumatic brain injury may lead to neurodegenerative disease. Rochoy *et al*’s study found intracranial hypertension was a possible association with AD [12]. So we have reasons to believe a pathophysiological hypothesis that traumatic brain injury and intracranial hypertension secondary to TBI may cause diffuse amyloid plaques to increase the risk of Alzheimer’s disease.

The positive result is consistent with the connection between traumatic brain injury and AD found in a meta-analysis [13]. The previous meta-analysis includes retrospective case-control studies and prospective cohort studies, but our study only pools prospective cohort studies. In contrast to retrospective case-control studies, prospective cohort studies have advantages in assessing a potential causal relationship between traumatic brain injury and AD, because all information about traumatic brain injury is collected before participants know whether they will develop AD.

In addition, Livingston *et al*’s [14] study estimated the RR of TBI of all severities for all cause dementia. After pooling RR, they found that all severity midlife traumatic brain injury were associated with all-cause dementia. Unlike their studies, subjects in our studies included young, middle-aged, and elderly TBI patients. And, we also conducted a subgroup analysis to compare the association between moderate and severe brain injury and Alzheimer’s disease.

When considering the severity of traumatic brain injury, eight studies defined the severity of traumatic brain injury. The relative risk of AD were higher in moderate and severe traumatic brain injury populations than in mild traumatic brain injury populations. The result was consistent with preconceived opinion that more severe injury induces more serious adverse events. And some pathological studies had found severe traumatic brain injury, and especially repeated mild traumatic brain injury can initiate long-term neurodegeneration processes leading to pathological features that have similarities with AD [15,16]. However, Li *et al*’s [17] study did not find significant association between severe traumatic brain injury and AD. This might be due to the limited number of studies that included in their study. The other reason was that previous study just considered traumatic brain injury with LOC as moderate and severe traumatic brain injury, but did not consider Glasgow Coma Scale score.

Although our results found that there was a significant association between risk of AD and traumatic brain injury, especially in moderate and severe traumatic brain injury populations, there was substantial statistical heterogeneity. This might be due to many included studies did not distinguish the severity of traumatic brain injury. Future high-quality, prospective studies would be beneficial to clarify the connection between the severity of traumatic brain injury and risk of AD.

### Limitations

Similar to other systematic reviews and meta-analyses, in our study there are substantial statistical and clinical heterogeneity. Different studies used a different way of characterizing traumatic brain injury exposure. Some were based on penetrating traumatic brain injury while others closed head injury. Little studies defined single and repetitive brain injury, So we can not estimate whether multiple TBIs increase the risk further. Also, the diagnostic criteria for Alzheimer’s disease are different in included studies. Some cognitive disorders might have been wrongly labelled Alzheimer’s disease.

In addition, in some studies subjects were older than 60 when they entered the cohort, which means participants had to have reached old age without having become demented in order to be included. Little studies reported the average delay between the TBI and the onset of the pathologies of AD.

Additionally, the definitions of injury severity were varied among the studies. While most studies considered changes such as drop in Glasgow Coma Scale (GCS) score, traumatic brain injury with LOC as moderate and severe traumatic brain injury, others did not report some of these outcomes.

### Conclusion

In summary, the results from this meta-analysis showed that Traumatic brain injury, especially moderate and severe traumatic brain injury may be associated with increased risk of AD.

## Supporting information

S1 Table Characteristics of the 17 studies included in the meta-analysis. (DOC)

S1 Appendix PRISMA 2009 checklist. (DOC)
