# Supplementary material for: A meta-analysis of cohort studies: Traumatic brain injury and risk of Alzheimer’s Disease
Source: PLoS One. 2021 Jun 22;16(6):e0253206. doi: 10.1371/journal.pone.0253206 (PMC8219123; doi:10.1371/journal.pone.0253206)
Supplement: S1 Table — (DOC) [file pone.0253206.s001.doc]

Supplementary table 1 characteristics of the 17 studies included in the meta-analysis

| **Reference** | **Study design** | **Region** | **Sample size** | **Average age (years)** | **Disease ascertainment** | **Years of follow-up** | **RR(95%CI)** | **Quality rating** |
| --- | --- | --- | --- | --- | --- | --- | --- | --- |
| Abner 2014 ^(1)^ | Cohort | The United States | 649 | 72.9 | CERAD | 15 | 1.47(1.03-2.09) | 9 |
| Abner 2014 ^(1)^ | Cohort | The United States | 649 | 72.9 | CERAD | 15 | 1.18(0.83-1.68) | 8 |
| Dams-O'Connor 2013 ^(2)^ | Cohort | The United States | 4225 | 74.9 | NINCDS-ADRDA | 6 | 0.95(0.65-1.38) | 8 |
| Fischer 2008 ^(3)^ | Cohort | Austria | 479 | 75.8 | NINCDS-ADRDA | 2.5 | 0.48(0.16-1.31) | 6 |
| Mehta 1999 ^(4)^ | Cohort | The Netherlands | 6645 | 68.9 | NINCDS-ADRDA | 2.1 | 0.80(0.40-1.90) | 8 |
| Nordstrom 2014 ^(5)^ | Cohort | Sweden | 811622 | 18.0 | ICD-9 | 33 | 1.00(0.50-2.00) | 8 |
| Plassman 2000 ^(6)^ | Cohort | The United States | 1776 | 72.9 | DSM-Ⅲ-R and NINCDS-ADRDA | 40 | 2.16(1.10-4.23) | 8 |
| Schofield 1997 ^(7)^ | Cohort | The United States | 271 | 75.3 | NINCDS-ADRDA | 5 | 1.05(0.34-3.22) | 6 |
| Wang 2012 ^(8)^ | Cohort | Taiwan | 269550 | 40.8 | ICD-9 | 5 | 1.49(1.08-2.07) | 7 |
| Tolppanen 2017^(9)^ | Cohort | Finland | 353581 | 63.0 | NINCDS-ADRDA and DSM-IV | 17 | 1.19(1.15-1.23) | 9 |
| Robinson 2019 ^(10)^ | Cohort | [The United Kingdom](javascript:;) | 110 | 66.2 | CERAD | 20 | 0.87(0.37-2.07) | 9 |
| Nemetz 1999^(11)^ | Cohort | The United States | 5023 | ≥40.0 | NINCDS-ADRDA | ≥10 | 1.20(0.80-1.70) | 8 |
| LoBue 2018 ^(12)^ | Cohort | The United States | 2719 | ≥50.0 | NINCDS-ADRDA | 4 | 0.80(0.63-1.01) | 9 |
| Fann 2018 ^(13)^ | Cohort | The United States | 2794852 | ≥50.0 | NINCDS-ADRDA | 14 | 1.16(1.12-1.22) | 8 |
| Gardner 2017 ^(14)^ | Cohort | The United States | 984 | 67.3 | MMSE | 22 | 1.28(1.01-1.61) | 8 |
| Weiner 2017 ^(15)^ | Cohort | The United States | 180 | 69.1 | MMSE | Not reported | 0.87(0.33-2.36) | 9 |
| Nordström 2018^(16)^ | Cohort | Sweden | 136233 | ≥50 | ICD-10 | 15.3 | 1.58(1.49-1.69) | 8 |

CERAD: The Consortium to Establish a Registry for Alzheimer's Disease. NINCDS-ADRDA: National Institute of Neurological and Communicative Diseases and Stroke- Alzheimer's Disease and Related Disorders Association. DSM-Ⅲ-R: Diagnostic and Statistical Manual of Mental Disorders, Third Edition Revised. ICD-9,10: International Classification of Diseases, Ninth Revision, Tenth Revision. DSM-IV: Diagnostic and Statistical Manual of Mental Disorders, Fourth Edition. MMSE: Mini-Mental State Examination test.

**References**

[1] Luchini C, Stubbs B, Solmi M, [Veronese](http://search.cnki.com.cn/Search/Result?author=Nicola Veronese) N. Assessing the quality of studies in meta-analyses: advantages and limitations of the Newcastle Ottawa Scale. World J Meta-Anal. 2017; 5: 80-84.

[2] Abner E L, Nelson P T, Schmitt F A, Browning S R, Fardo D W, Wan L, et al. Self-reported traumatic brain injury and risk of late-life impairment and AD pathology in an AD center cohort. Dement Geriatr Cogn. 2014; 37: 294-306.

[3] Dams-O'Connor K, Gibbons L E, Bowen J D, McCurry S M, Larson E B, Crane P K. Risk for late-life re-injury, dementia and death among individuals with traumatic brain injury: a population-based study. J Neurol Neurosurg Psychiatry. 2013; 84: 177-182.

[4] Mehta K M, Ott A, Kalmijn S, Slooter A J C, Van Duijn C M, Hofman A, et al. Head trauma and risk of dementia and Alzheimer’s disease: The Rotterdam Study. Neurology. 1999; 53: 1959-1959.

[5] Nordström P, Michaëlsson K, Gustafson Y, Nordström A. Traumatic brain injury and young onset dementia: a nationwide cohort study. Ann Neurol. 2014; 75: 374-381.

[6] Plassman B L, Havlik R J, Steffens D C, Helms M J, Newman T N, Drosdick D, et al. Documented traumatic brain injury in early adulthood and risk of Alzheimer’s disease and other dementias. Neurology. 2000; 55: 1158-1166.

[7] Schofield P W, Tang M, Marder K, Bell K, Dooneief G, Chun M, et al. Alzheimer's disease after remote traumatic brain injury: an incidence study. J Neurol Neurosur Ps. 1997; 62: 119-124.

[8] Wang H K, Lin S H, Sung P S, Wu M H, Hung K W, Wang L C, et al. Population based study on patients with traumatic brain injury suggests increased risk of dementia. J Neurol Neurosur Ps. 2012; 83: 1080-1085.

[9] Tolppanen A M, Taipale H, Hartikainen S. Head or brain injuries and Alzheimer's disease: A nested case-control register study. Alzheimer's Demen. 2017; 13: 1371-1379.

[10] Robinson A C, Davidson Y S, Horan M A, Cairns M, Pendleton N, Mann D M. No association between traumatic brain injury with loss of consciousness and Alzheimer disease pathology—Findings from the University of Manchester Longitudinal Study of Cognition in Normal Healthy Old Age. Int J Geriatr Psych. 2019; 34:1262-1266.

[11] Nemetz P N, Leibson C, Naessens J M, Beard M, Kokmen E, Annegers J F, et al. Traumatic brain injury and time to onset of Alzheimer's disease: a population-based study. Am J Epidemiol. 1999; 149: 32-40.

[12] LoBue C, Woon F L, Rossetti H C, Hyna L S, Hart Jr J, & Cullum C M. Traumatic brain injury history and progression from mild cognitive impairment to Alzheimer disease. Neuropsychology. 2018; 32: 401-409.

[13] Fann J R, Ribe A R, Pedersen H S, Fenger-GrÃ¸n M, Christensen J, Benros M E, et al. Long-term risk of dementia among people with traumatic brain injury in Denmark: a population-based observational cohort study. Lancet Psychiat. 2018; 5: 424-431.

[14] Gardner R C, Langa K M, Yaffe K. Subjective and objective cognitive function among older adults with a history of traumatic brain injury: A population-based cohort study. PLoS Med. 2017; 14: e1002246.

[15] Weiner M W, Harvey D, Hayes J, Landau S M, Aisen P S, Petersen R C, et al. Effects of traumatic brain injury and posttraumatic stress disorder on development of Alzheimer's disease in Vietnam veterans using the Alzheimer's Disease Neuroimaging Initiative: preliminary report. Alzheimer's Demen. 2017; 3: 177-188.

[16] Nordström A, Nordström P. Traumatic brain injury and the risk of dementia diagnosis: A nationwide cohort study[J]. PLoS medicine, 2018, 15(1): e1002496.
